# Supplementary figures and images for: Serology suggests adequate safety measures to protect healthcare workers from COVID-19 in Shiga Prefecture, Japan
Source: PLoS One. 2022 Jun 24;17(6):e0270334. doi: 10.1371/journal.pone.0270334 (PMC9231724; doi:10.1371/journal.pone.0270334)

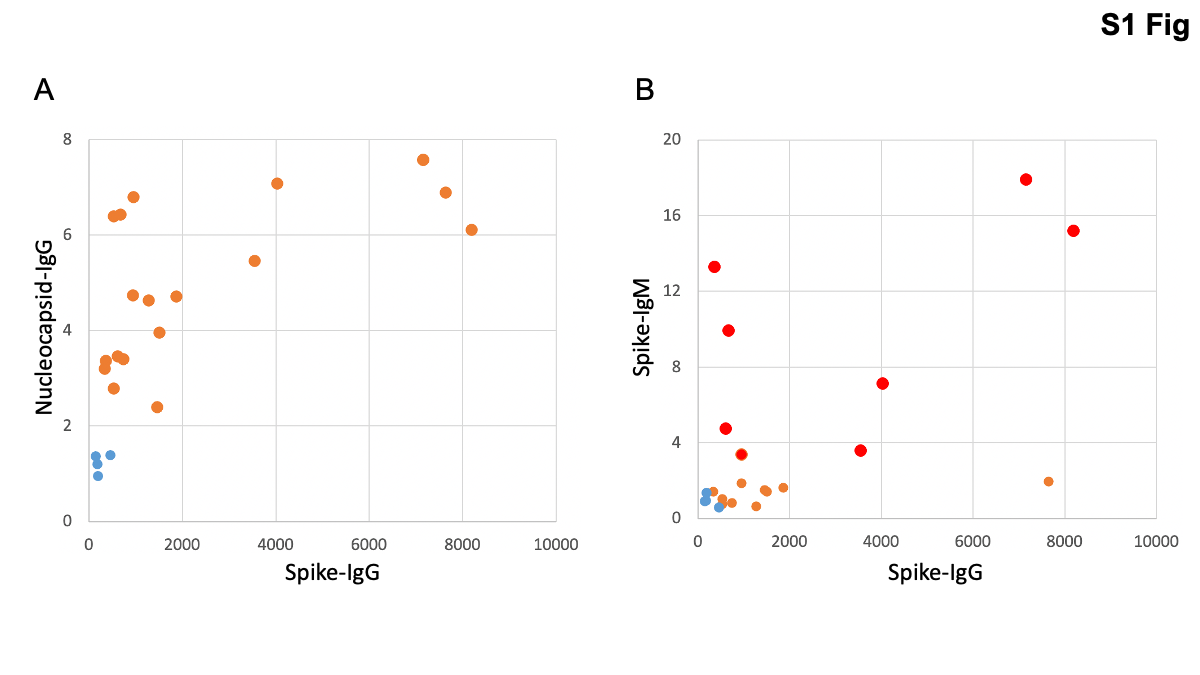

Supplement: S1 Fig — (TIFF) [file pone.0270334.s001.tiff]

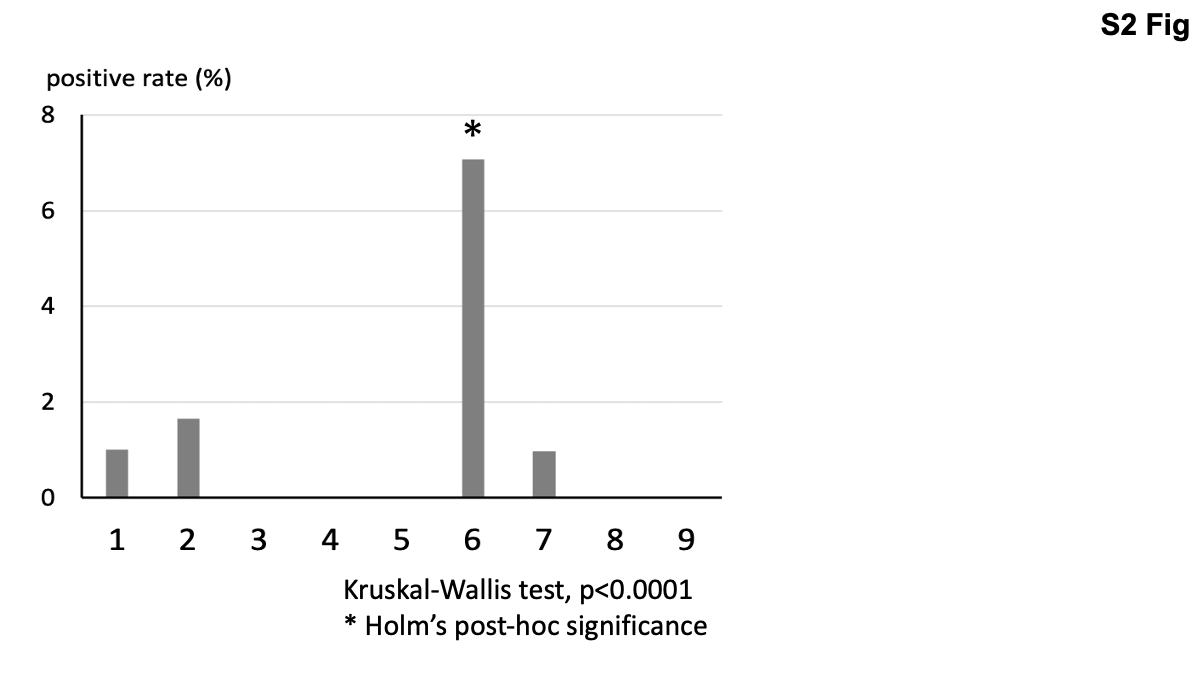

Supplement: S2 Fig — (TIFF) [file pone.0270334.s002.tiff]

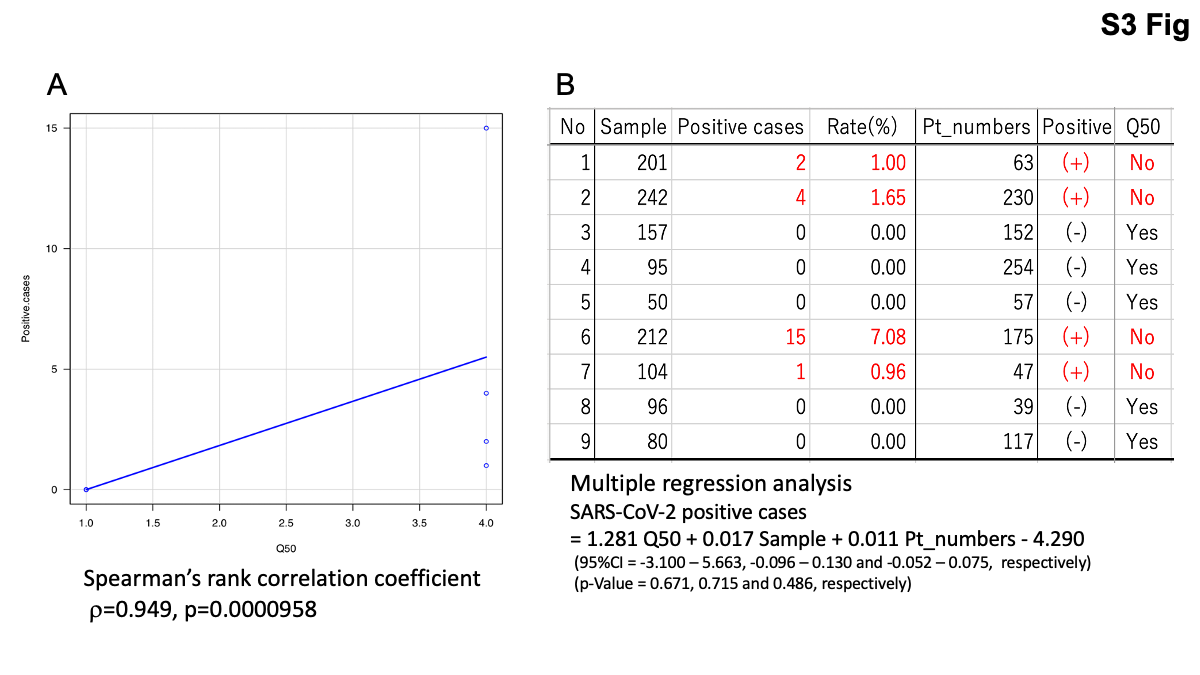

Supplement: S3 Fig — (TIFF) [file pone.0270334.s003.tiff]
