# Supplementary material for: Serology suggests adequate safety measures to protect healthcare workers from COVID-19 in Shiga Prefecture, Japan
Source: PLoS One. 2022 Jun 24;17(6):e0270334. doi: 10.1371/journal.pone.0270334 (PMC9231724; doi:10.1371/journal.pone.0270334)
Supplement: Attachment — Submitted filename: Reviewer_comment.docx [file pone.0270334.s008.docx]

**Reviewer’s report**

**Title:** Serology suggests adequate safety measures to protect healthcare workers from

COVID-19 in Shiga Prefecture, Japan

**Version: Date**: 9^th^ December 2021

**Reviewer:** Samuel Bosomprah

**Reviewer’s report**

The authors stated that this was a seroprevalence study aimed “to estimate the timing of HCWs’ exposure to SARS-CoV-2 and to analyse whether the HCWs had been exposed to SARSCoV-2 earlier than the general population as well as to determine whether the seroprevalence of HCWs was higher than that of the general population in Japan”

**General comment:**

The manuscript did not read well as a research document. The language is not consistent with a research paper write up. For example, the authors used terms such as “noted” in the introduction instead of, say, cases were “recorded” etc. Another term was “clarify” in method section “To clarify whether the questionnaire responses for each individual HCW were predictive of SARS-CoV-2 antibody seropositivity…” and “To clarify whether the questionnaires from each institute’s representative were predictive of hospitals where there were seropositive HCWs…” These are not appropriate statistical terminologies.

**Introduction**

- I would have expected the authors to begin the introduction with clear statement of the problem before elaborating on it in terms of the magnitude of the problem and which group is unfairly or disproportionately affected.
- The authors did not provide compelling argument for why the study is important (i.e., justification/significance/rationale) and what the knowledge/research gap is, which they are seeking to fill in

**Methods**

- The method section is not well structured. The authors should consider renaming “study setting” as “Study design and participants” and integrate the “study participants” texts.
- Authors should avoid the phrase “…investigation prospectively recruited..” for a cross-sectional study design
- Authors should consider integrating the subheadings “Evaluation of individual personal protective equipment (PPE)” and “Evaluation for institutional infection prevention and control (IPC)” into “Data analysis” and edit the language to read well.
- Authors did not provide the assumptions on arriving at a sample size of 1237. They should consider including a section on “sample size consideration”, which should describe how the sample size of 1237 was arrived at.
- The section “Patient and public involvement” should be renamed as “Ethical statement” and should be edited accordingly.

**Findings**

- “Findings” should be edited to “Results”
- The analysis and presentation of results did not meet the statistical rigour required of a seroprevalence study. The tables were poorly presented – only frequencies were presented; no corresponding proportions (seroprevalence) were presented (See tables 1, 2 and 3). P-values were inappropriately presented. It is recommended to present p-values to 3 decimal places.

**Discussion**

- The authors were inappropriately referencing tables and figure as if they are writing up results
- The authors should focus on interpreting the results instead of rehashing same in the discussion.
- Should discuss the strength and limitations of the study.
- They should also discuss the impact of the findings as well as prescription of future work.
- No apparent conclusion

**Discretionary Revisions:** Reject

**Level of interest:** An article of importance in its field

**Quality of written English:** Authors should proofread and correct for grammatical errors and use appropriate research language

**Statistical review:** No, I am a statistician and have reviewed the statistical methods used.

**Declaration of competing interests:** I declare that I have no competing interest
