# Supplementary material for: Serology suggests adequate safety measures to protect healthcare workers from COVID-19 in Shiga Prefecture, Japan
Source: PLoS One. 2022 Jun 24;17(6):e0270334. doi: 10.1371/journal.pone.0270334 (PMC9231724; doi:10.1371/journal.pone.0270334)
Supplement: Attachment — Submitted filename: PtoP_PLosOne.docx [file pone.0270334.s009.docx]

**Response to Reviewers**

Reviewer #1

Major comments

1) Abstract.

Please specify P-value and the regression coefficient.

Following your suggestion, we’ve added p- & β-Values of each variable, additionally into Table 2-3.

2) Methods.

2.1. The use of logistic regression models requires the verification of some assumptions.

As you have suggested, if the seroprevalence has been 1-2% and we've analyzed 8 variables, the logistic regression will require about 4000-8000 cases. Thus, binomial logistic regression was initially performed using the stepwise variable reduction method using p-Value. However, in this cohort, referring to the seroprevalence and number of participants (1.78% and 1237 cases), the confirmatively regression analysis was conducted with only 2 variables, N95 mask implementation and working period in the red zone (Page 7, Line 137 - 141).

This study limitation has also been described in the revised manuscript (Page 18, Line 371 - 382).

2.2. I suggest that the authors briefly address this aspect in the manuscript, motivating their choices to use non-parametric tests.

Only 9 institutes were included in this study, meaning small number. In addition, both the seropositive case numbers and rates of each institute were out of normal distribution through Shapiro-Wilk normality test (p=0.00006657 and 0.00007803), respectively. Thus, in order to evaluate the difference and questionnaire of each institution, we applied nonparametric measures such as the Kruskal-Wallis test and Spearman’s rank correlation coefficient, respectively, to the analyses.

These are described in the revised manuscript (Page 7 - 8, Line 141 - 147).

2.3. Dear authors, I ask if the possibility to test for internal consistency of questionnaires has been evaluated.

Under the health & infection management of Shiga prefecture administration, the zone management and questionnaire investigation were similarly conducted in all the participated hospitals (Page 4, Line 80 - 83).

In addition, these questionnaires were designed, referring also to the checklists of Japanese society for infection prevention and control and of national institute of infectious diseases (Page 5, Line 99 - 101).

3) Results.

3.1. I suggest speaking of "more and less significant" or "high and low significance" rather than "significant and non-significant."

3.2. I suggest commenting on the results also based on the intensity of the phenomenon.

Following your suggestion, we have modified our descriptions into "highly significant" and "weak association," etc.

4) Discussion.

4.1. I suggest making a comparison with the literature published on this topic.

Based on your suggestion, we’ve compared with the other literatures and produced more fruitful discussion (Page 16 -17, Line 344 - 350 and 357 - 358).

4.2. I suggest clearly specifying the limitations of the study.

According to your suggestion, we have described some limitations of this study, and produced a more balanced work (Page 18, Line 371 - 389).

Minor comments

m1) References. Regarding websites, I suggest specifying the name of the source and providing an access date.

According to your suggestion, we have added the information into References.

Reviewer #2

General comment: The language is not consistent with a research paper write up.

Base on your suggestion, we have used adequate terminologies in the sections of “Introduction” and “Methods”.

Introduction

• The authors to begin the introduction with clear statement of the problem.

• The authors did not provide compelling argument for why the study is important

Considering your suggestion, we have reconstructed the “Introduction” section better.

Methods

• “Study design and participants”

• Authors should avoid the phrase “…investigation prospectively recruited...”

• “Data analysis”

• “Ethical statement”

Following your suggestion, we have reconstructed the “Methods” section better.

• Authors did not provide the assumptions on arriving at a sample size of 1237.

We appreciate your meaningful suggestion. As you have suggested, if the seroprevalence has been 1-2% and we've analyzed 8 variables, the logistic regression will require about 4000-8000 cases. Thus, binomial logistic regression was initially performed using the stepwise variable reduction method using p-Value. However, in this cohort, referring to the seroprevalence and number of participants (1.78% and 1237 cases), the confirmatively regression analysis was conducted with only 2 variables, N95 mask implementation and working period in the red zone (Page 7, Line 137 - 141).

In addition, the study limitation has been described in the revised manuscript (Page 18, Line 371 - 382).

Findings

• should be edited to “Results”

• Tables were poorly presented – only frequencies were presented; no corresponding proportions (seroprevalence) were presented (See tables 1, 2 and 3). P-values were inappropriately presented. It is recommended to present p-values to 3 decimal places.

We appreciate your meaningful suggestion, and have reconstructed all the tables.

Discussion

• The authors were inappropriately referencing tables and figure as if they are writing up results

• The authors should focus on interpreting the results instead of rehashing same in the discussion.

• Should discuss the strength and limitations of the study.

• They should also discuss the impact of the findings as well as prescription of future work.

• No apparent conclusion

Base on your fruitful suggestions, we have reconstructed the “Discussion” section better. We have also described the study limitations and prescription in future (Page 18, Line 371 - 382 and 387 - 389).

We’ve added apparently the “Conclusion” section in the revised manuscript.

Reviewer #3

- What is the sensitivity and specificity of the kit used to past covid exposure?

In this context, referring to the manufacturer's’ specification documents and previously reports, we have clarified them in the “Method” section (Page 6, Line 116 - 118).

- Do we have any supporting evidence on how the seropositivity behaves over time? Can exposure to covid19 be perfectly covered by the seropositivity?

- How the seropositivity behaves over time? Can exposure to covid19 be perfectly covered by the seropositivity?

In a healthy adult population (, not in children), the seropositivity has almost completely reflected the previous infections. Additionally, the time courses of each antibody titer decay have been in line with the previous reports. In the revised version, we have described them in the “Result” section (Page 9, Line 164 - 167).

- Covid19 variants are also time dependent. Can this be taken into account in the current model? If not, how do you explain the validity of the model when lacking such data?

The survey was performed before alpha-variant predominant expansion in Japan, and the results might not reflect that in the predominant period of alpha, delta, or Omicron variant, etc. Nevertheless, the implementation procedure of PPE and IPC is likely similar to that of the original SARS-CoV-2, so the study data will contribute to the preventive measures of the present and future variants in healthcare facilities.

We have described this issue in the study limitations (Page 18, Line 371 - 382).

- Removal of the terminal care unit of one hospital in which the outbreak took place from the analysis is problematic.

Following your suggestion, we have evaluated even both models included with or without the specific hospital where the outbreak took place (Table 1; Page 10, Line 194 - 205).

- It is not clear whether the fitted binomial logistic regression analysis was a multivariable model? If so this should be made clear and should be explained what method was used to retain those 3 variables. Was it simply variables retained in the model which had significant p- values? Was there kind or model selection such as backward, forward or stepwise attempted?

As you suggested, we clarified that binomial logistic regression was initially performed using the stepwise variable reduction method using p-Value. In this cohort, referring to the seroprevalence and number of participants (1.78% and 1237 cases), the confirmatively regression analysis was conducted with only 2 variables, N95 mask implementation and working period in the red zone.

These are described in the "Statistical analysis” of the “Method” section (Page 7, Line 137 - 141).

- The article should highlight some restrictions of the study as highlighted above. This should be done in the discussion.

As you suggested, we have highlighted the study limitations in the “Discussion” section (Page 18, Line 371 - 382).
