# Supplementary material for: Serology suggests adequate safety measures to protect healthcare workers from COVID-19 in Shiga Prefecture, Japan
Source: PLoS One. 2022 Jun 24;17(6):e0270334. doi: 10.1371/journal.pone.0270334 (PMC9231724; doi:10.1371/journal.pone.0270334)
Supplement: Attachment — Submitted filename: Reviewer_comment_round 2.docx [file pone.0270334.s010.docx]

**Reviewer’s report**

**Title:** Serology suggests adequate safety measures to protect healthcare workers from

COVID-19 in Shiga Prefecture, Japan

**Version 2: Date**: 30^th^ April 2022

**Reviewer:** Samuel Bosomprah

**General comment:**

The authors addressed some of my earlier comments. But there remains some major work especially presentation of results and tables. The specifics are stated below

**Methods**

- Authors should state the eligibility (inclusion and exclusion) criteria under the subsection “Study design and participants”
- “Data analyses” and Statistical analysis” sections should be integrated into one subheading called “Statistical analysis”.
- Authors did not provide the assumptions on arriving at a sample size of 1237. They should consider including a section on “sample size consideration”, which should describe how the sample size of 1237 was arrived at.

**Results**

- The results section should start with the subheading “Characteristics of study participants”. This section should have a table and comments describing who these participants were: example, distribution of sex, age, and other sociodemographic characteristics…
- The analysis and presentation of results did not meet the statistical rigour required of a seroprevalence study. The tables were poorly presented. P-values were inappropriately presented. It is recommended to present p-values to 3 decimal places.
- The betas in the logistic regression table are uninterpretable. Odds ratio would have been better…

**Discretionary Revisions:** Reject

**Level of interest:** An article of importance in its field

**Quality of written English:** Authors should proofread and correct for grammatical errors and use appropriate research language

**Statistical review:** No, I am a statistician and have reviewed the statistical methods used.

**Declaration of competing interests:** I declare that I have no competing interest
