# Supplementary material for: Serology suggests adequate safety measures to protect healthcare workers from COVID-19 in Shiga Prefecture, Japan
Source: PLoS One. 2022 Jun 24;17(6):e0270334. doi: 10.1371/journal.pone.0270334 (PMC9231724; doi:10.1371/journal.pone.0270334)
Supplement: Attachment — Submitted filename: PtoP_PLosOne2.docx [file pone.0270334.s011.docx]

**For Academic Editor**

• Please reconsider the use of the term "discrimination" and the presentation of your conclusions.

According to your suggestion, we've changed the word “discriminate” to “consider” or “recognize”.

**Response to Reviewers**

Reviewer #1

1) Logistic regression assumptions are still not discussed; multicollinearity should be added to the publication.

We’ve taken your advice and clarified variance inflation factor (VIF) of each variable has been used for evaluating the risk of multicollinearity in "Statistical analyses" section (Page 7, Line 132-133).

Additionally, in the binomial logistic regression model with only 2 variables, N95 mask implementation and working period in the red zone, we’ve clarified those VIFs (Page 12, Line 233-234).

Meanwhile, also referring the suggestion of Reviewer 2, in the re-revise version of our manuscript, Tables 2-3 have been re-summarized with odds ratio rather than beta, etc.

Reviewer #2

Methods

• Authors should state the eligibility (inclusion and exclusion) criteria under the subsection “Study design and participants”.

We’ve taken your advice and added the rationale for the provision for red zone workers into the "Study design and participants" section (Page 5, Line 91-93).

• “Data analyses” and Statistical analysis” sections should be integrated into one subheading called “Statistical analysis”.

Following your suggestion, we have integrated these two into one section " Statistical analysis".

• Authors should provide the assumptions on arriving at a sample size of 1237.

We’ve taken your advice and added the rationale for the size of the recruited participants into the "Study design and participants" section (Page 5, Line 85-88).

Results

• The results section should start with the subheading “Characteristics of study participants”. This section should have a table and comments describing who these participants were: example, distribution of sex, age, and other sociodemographic characteristics…

In fact, the other characteristics of the present study participants couldn't be precisely clarified, because individual questionnaire didn't include such questions. However, the second serological survey of HCWs was similarly conducted December 2021 in Shiga prefecture. In the second survey, 1600 HCWs was characterized with age of 42.1 ±16.4 (indicating mean ±S.D. below) years old, body mass index of 23.4 ±8.7 kg/m^2^, and 66.2% of female predominance. Thus, the present study was presumably composed with similar character population.

These have been described in the re-revised manuscript (Page 8, Line 164 – Page 9, Line 170).

• The analysis and presentation of results did not meet the statistical rigor required of a seroprevalence study. The tables were poorly presented. P-values were inappropriately presented. It is recommended to present p-values to 3 decimal places.

• The betas in the logistic regression table are uninterpretable. Odds ratio would have been better…

According to your suggestions, we have summarized and refined Tables 2-3.
